# B-type natriuretic peptide and estimated glomerular filtration rate at ICU admission as a predictor of delirium

**DOI:** 10.1186/cc14556

**Published:** 2015-03-16

**Authors:** T Hirayama, S Ichiba, K Sato, T Yumoto, K Tsukahara, M Terado, U Yoshihito, T Ugawa

**Affiliations:** 1 Okayama University Hospital, Okayama, Japan

## Introduction

Delirium in the ICU is a predictor of mortality and cognitive impairment at hospital discharge. Although several pathways for delirium have been described, it is very difficult to predict the occurrence of delirium. In this study, we examined plasma biomarkers in delirious and nondelirious patients at admission and whether the biomarkers can predict onset of delirium.

## Methods

We targeted 103 ICU patients in Okayama University Hospital between April 2013 and February 2014. Delirium was diagnosed using the Confusion Assessment Method - ICU. On admission, blood was obtained for biomarker analysis. Patients with severe head injury and under 16 years old were excluded. *P *< 0.05 was considered statistically significant.

## Results

Thirty-seven delirious and 66 nondelirious patients were included. We found that delirious patients tented to have higher B-type natriuretic peptide (BNP) levels and to have lower estimated glomerular filtration rate (eGFR) (BNP: delirious patients 188.6 pg/ml, nondelirious patients 78.2 pg/ml (*P *= 0.001); eGFR: delirious patients 58.6 ml/minute/1.73 m^2^, nondelirious patients 81.3 ml/minute/1.73 m^2 ^(*P *= 0.020)). Procalcitonin (PCT) and D-dimer were almost the same between delirious and nondelirious patients (PCT: delirious patients 0.202 ng/ml, nondelirious patients 0.150 ng/ml (*P *= 0.613); D-dimer: delirious patients 5.25 ng/ml, nondelirious patients 5.35 ng/ml (*P *= 0.714)).

## Conclusion

BNP and eGFR in ICU admission was associated with delirium. PCT and D-dimer in ICU admission was not associated with delirium. BNP and eGFR might evaluate a predictor of delirium in ICU.
